# Assessment of Residual Cortical Function by Using Tc-99m DMSA SPECT at Follow-Up in Non-Operatively Treated Patients with Traumatic Renal Injuries: A Prospective Single-Centre Study

**DOI:** 10.3390/jcm14176276

**Published:** 2025-09-05

**Authors:** Seong Hyeon Yu, Taek Won Kang, Chan Park, Sang-Geon Cho

**Affiliations:** 1 Department of Urology, Chonnam National University Medical School, Chonnam National University Hospital, Gwangju 61469, Republic of Korea; domer12@hanmail.net (S.H.Y.); sydad@hanmail.net (T.W.K.); 2 Department of Radiology, Chonnam National University Medical School, Chonnam National University Hospital, Gwangju 61469, Republic of Korea; rdparkchan@gmail.com; 3 Department of Nuclear Medicine, Chonnam National University Medical School, Chonnam National University Hospital, Gwangju 61469, Republic of Korea

**Keywords:** renal injury, kidney function tests, Tc-99m dimercaptosuccinic acid, single-photon emission computed tomography, therapeutic embolisation

## Abstract

**Background/Objectives**: This prospective study aimed to assess residual cortical function at follow-up in patients with traumatic renal injuries using Tc-99m dimercaptosuccinic acid (DMSA) single-photon emission computed tomography (SPECT) and evaluate clinical factors associated with residual cortical function. **Methods**: A total of 59 patients with renal injury who were treated non-operatively and underwent Tc-99m DMSA SPECT at the follow-up (3 months ± 1 year) were enrolled. The correlation between residual cortical function and renal injury grades, alongside other clinical factors, was analysed. **Results**: The mean age of the patients was 49.10 ± 22.67 years, and 35 (59.3%) were male. In total, 28 patients (47.5%) had high-grade injuries, and 20 (33.9%) underwent a renal artery endovascular procedure (RAE). High-grade renal injury correlated with laboratory renal function and DMSA scintigraphic parameters, especially SPECT split renal function (SRF) (ρ = −0.565; *p* < 0.001); meanwhile, a significant decrease existed in DMSA scintigraphic parameters in patients with high-grade injuries. Furthermore, laboratory renal function and DMSA scintigraphic parameters were significantly decreased in patients who underwent RAE. The multivariable analysis highlighted that high grade renal injury (odds ratio [OR], 9.50; 95% confidence interval (CI), 1.78–50.61; *p* = 0.008) and RAE (OR, 5.15; 95% CI, 1.07–24.88; *p* = 0.041) were significant factors associated with decreased residual cortical function. **Conclusions:** Tc-99m DMSA SPECT provides accurate information on the residual cortical function at follow-up in patients with renal injuries. Additionally, high-grade renal injury and RAE were associated with decreased residual cortical function.

## 1. Introduction

Traumatic renal injury is fairly common, accounting for up to 5% of all trauma cases and 65% to 90% of urogenital trauma [1,2]. The mechanisms involved in renal injury are diverse and result in the direct destruction of the kidney and/or hilar structures. The most common mechanism is blunt trauma from traffic accidents, falls, sporting injuries, or assault, while penetrating injuries and iatrogenic injuries have been presented in up to 20% of cases [2,3]. In addition, renal injury is often associated with significant injuries in polytraumatised patients, with a wide range of morbidity and mortality [2]. Hence, a multidisciplinary approach including trauma surgeons, urologists, and interventional radiologists is essential to improve short- and long-term outcomes in patients with renal injury.

Over the last few decades, the management of traumatic renal injuries has undergone a paradigm shift toward non-operative management (NOM). Notably, NOM has subsequently become the standard treatment in patients with low-grade renal injuries and hemodynamically stable high-grade renal injuries [2,4,5]. Furthermore, NOM has recently been encouraged for the ultimate objective of renal preservation even in patients with hemodynamically unstable high-grade renal injuries [5]. In particular, the renal artery endovascular procedure (RAE) plays a crucial role in increasing the success rate of NOM in most patients [6]. However, despite the efficacy of RAE in bleeding control, this procedure is inevitably accompanied by some risks, including renal parenchymal infarction and contrast agent nephrotoxicity [7]. Therefore, short- and long-term outcomes of traumatic renal injuries remain heterogeneous, mostly based on retrospective data and assessments of renal function using serum creatinine and glomerular filtration rate (GFR) [6,8,9,10]. Moreover, the actual cortical damages from traumatic renal injuries and associated treatments (e.g., RAE) have not been elucidated, and guideline recommendations for assessing post-traumatic renal cortical function are diverse [5]. Thus, the lack of information on post-traumatic residual renal cortical function may result in patient anxiety due to an unknown risk of renal failure, especially in patients with high-grade renal injuries who underwent RAE.

To date, efforts are being made to quantitatively assess post-traumatic residual renal cortical function using Tc-99m dimercaptosuccinic acid (DMSA) scintigraphy [11,12,13]. DMSA scintigraphy can provide relatively accurate information on residual functional integrity; however, this technique has a limitation in that it relies on split renal function (SRF), which can only detect a relative decrease in cortical integrity of the injured kidney compared to the contralateral, uninjured kidney. Hence, DMSA scintigraphy may be inappropriate for assessing the absolute amount of residual functional cortex following renal injury.

In contrast, DMSA single-photon emission computed tomography (SPECT) has advantages over planar imaging in DMSA scintigraphy, offering improved image resolution and quantification through the provision of three-dimensional (3D) volumetric indices and quantitative parameters of renal cortical integrity [14]. Notably, our previous study clarified the additional value of DMSA SPECT over planar imaging in providing more clinically relevant and comprehensive information for the follow-up assessment of residual cortical function in patients with renal injuries [15]. Therefore, this study aimed to assess residual cortical function at follow-up in patients with renal injuries who underwent NOM treatment using Tc-99m DMSA SPECT and evaluate clinical factors associated with decreased residual cortical function.

## 2. Materials and Methods

### 2.1. Patients and Study Design

The grade of renal injury was classified based on the CT scan results according to the American Association for the Surgery of Trauma (AAST) injury scale [16], with high-grade renal injury defined as AAST grade 4 or higher. The management strategies were determined through a multidisciplinary approach, involving trauma surgeons, urologists, and interventional radiologists, based on contemporary guideline recommendations [2,4,5]. Patients who underwent NOM as initial treatment were included in the present study. Patients were excluded if they exhibited any of the following criteria: (1) underwent nephrectomy for initial treatment, (2) end-stage renal disease with or without haemodialysis, (3) follow-up loss, and (4) lack of medical records for assessing residual cortical function. A total of 59 renal injuries were found eligible for the final analysis.

### 2.2. Non-Operative Management for Renal Injury (Indications and Technique of RAE)

Hemodynamic stability was the primary criterion for managing renal injuries. Hemodynamically stable patients were managed using the NOM approach, which included bed rest, serial blood tests, transfusions, regular observation, and re-imaging. However, RAE was performed if the patient had active renal bleeding signs on the initial CT scan (e.g., intravascular contrast extravasation, pseudoaneurysms, arteriovenous fistula, or large perirenal haematoma) and needed significant transfusions without other major visceral injuries. The patients who failed NOM due to an unresponsiveness to fluid resuscitation, hypotension, or persistent gross haematuria also underwent RAE. Furthermore, even in hemodynamically unstable patients without evidence of other major visceral injuries requiring surgical exploration, NOM with RAE was performed as an initial treatment according to the contemporary guideline recommendations [2,4,5].

RAE was performed via a transfemoral approach, with a 6Fr vascular sheath placed, followed by aortography. Selective renal artery angiography was then performed, and superselective renal artery angiography was obtained just proximally to the injured renal artery segment. Superselective embolisation was performed using a microcoil to maximise the preservation of renal parenchyma. However, if the superselective embolisation failed due to the presence of a large aneurysm located at the distal portion of the main renal artery, proximal renal artery embolisation was performed using a vascular plug. In addition, endovascular revascularisation with or without a stent was performed in the case of renal artery occlusion.

### 2.3. Data Collection and Assessment of Residual Cortical Function

A detailed medical history was obtained from the medical records for the initial assessment of each patient at the time of renal injury, including age, body mass index (BMI), sex, diabetes mellitus, hypertension, underlying renal diseases, renal injury information (e.g., cause, injured kidney and AAST grades), concomitant organ injuries, RAE (performed or not), serum creatinine and GFR. The patients who underwent NOM were referred to a urologist for regular follow-ups. The follow-up studies, including serum creatinine, GFR and DMSA with SPECT imaging, were performed 3 months after the initial renal injury. In addition, patients with high-grade renal injuries underwent the same follow-up studies at 1 year after the renal injury.

For planar images, the photon counts in the anterior and posterior images were averaged by calculating the geometric mean of each kidney. The planar SRF of the injured kidney was calculated as the proportion of the geometric mean of the injured kidney divided by the geometric mean of both kidneys [15]. The renal contours were automatically delineated for SPECT images as 40% of the maximum uptake [17] using a dedicated nuclear imaging analysis software (MIM Maestro ver. 7.2, MIM Software Inc., Cleveland, OH, USA). The photon counts within the renal contours and the corresponding cortical volume were measured for each kidney to calculate SRF. As a per-patient analysis, the injured renal cortical volume (ICV) was measured as the volume of the delineated renal cortex of the injured kidney. These values were divided by body surface area to normalise interindividual variation to obtain the ICV index [15]. Absolute volumetric renal DMSA uptake (% injected dose) [18] was quantified for the injured kidney (injured renal cortical uptake, ICU) with decay correction, using the Q-Volumetrix MI software package on Xeleris V (GE Healthcare, Chicago, IL, USA).

### 2.4. Statistical Analysis

Statistical analysis was performed using STATA version 16.1 (StataCorp., College Station, TX, USA). A descriptive analysis was performed to assess patient demographics. Continuous variables are presented as the mean and standard deviation, and categorical variables are presented as frequencies (%). The correlation between AAST renal injury grade and clinical features, as well as DMSA scintigraphic parameters, was analysed using Pearson’s correlation analysis. Clinical features and DMSA scintigraphic parameters were compared according to the AAST renal injury grade (low vs. high) and RAE (performed or not) using the Wilcoxon rank-sum test, Student’s *t*-test, Pearson chi-square test, and Fisher’s exact test. A logistic regression test was performed to identify clinical factors associated with significantly decreased residual cortical function. In addition, clinical features and DMSA scintigraphic parameters in patients with high-grade renal injury were compared according to the follow-up period (3 months vs. 1 year). A *p*-value less than 0.05 was considered statistically significant.

### 2.5. Ethics Statement

The study protocol was approved by the Institutional Review Board (IRB) of Chonnam National University Hospital Research Institute of Clinical Medicine (IRB approval No. CNUH-2022-101). This study was conducted in accordance with the Declaration of Helsinki and the Ethical Guidelines for Clinical Studies, and the collected data were used in the study after obtaining informed consent from each patient.

## 3. Results

Data were collected for 59 cases of renal injury in this prospective observational study. The characteristics of patients with renal injury are summarised in Table 1. The mean age and BMI of the patients were 49.10 ± 22.67 years and 24.24 ± 4.22 kg/m^2^, respectively. A total of 35 (59.3%) patients were male. In addition, 16 (27.1%) patients had underlying renal cysts, and 10 (10.9%) had other underlying renal diseases, such as stone, vascular anomaly, or benign tumours. Regarding causes of renal injuries, traffic accidents (39.0%) were the most common cause, followed by falls (20.3%) and iatrogenic injury (20.3%). In terms of injury characteristics, left kidney injuries were observed in 35 (59.3%) patients, and high-grade renal injuries were observed in 28 (47.5%) patients. In addition, 26 (44.1%) patients had concomitant organ injuries such as the liver, spleen, adrenal gland, pancreas, and intestine. In total, 20 (33.9%) of the 59 patients with renal injury underwent renal artery endovascular procedure, including superselective renal artery embolisation (17, 28.8%), proximal renal artery embolisation (1, 1.6%), and endovascular revascularisation (2, 3.4%). Compared to the time of the index injury, laboratory renal function was significantly improved at follow-up, as indicated by serum creatinine (0.97 ± 0.39 vs. 0.81 ± 0.26) and GFR levels (88.87 ± 32.65 vs. 100.00 ± 30.86). On the DMSA scintigraphic parameters at the follow-up period (3 months), the planar SRF, SPECT SRF, ICV, ICU, and ICV index values were 44.39 ± 14.17%, 41.50 ± 14.73%, 93.65 ± 44.21 mL, 11.42 ± 5.49%, and 53.51 ± 23.14 mL/m^2^, respectively.

The renal injury grade correlated with clinical features (serum creatinine, GFR, and concomitant organ injuries) and DMSA scintigraphic parameters; specifically, the best correlation was observed with SPECT parameters (SRF, ICV, ICU, and ICV index; *p* < 0.001) (Table 2). Regarding comparisons of clinical features and DMSA scintigraphic parameters according to the AAST renal injury grade, patients with a high-grade renal injury possessed significantly higher serum creatinine levels (0.74 ± 0.19 vs. 0.89 ± 0.30; *p* = 0.024) at follow-up, and significantly lower planar SRF (48.75 ± 8.24 vs. 39.57 ± 17.61; *p* = 0.022), SPECT SRF (48.91 ± 6.38 vs. 33.30 ± 16.97; *p* < 0.001), ICV (113.07 ± 30.69 vs. 72.15 ± 47.32; *p* < 0.001), ICU (13.41 ± 3.38 vs. 9.22 ± 6.52; *p* = 0.004), and ICV index (64.58 ± 12.55 vs. 41.27 ± 26.06; *p* < 0.001) values (Table 3). However, there was no additional deterioration 1 year after the time of renal injury, even in patients with high-grade renal injury (Table 4).

**Figure 1:**
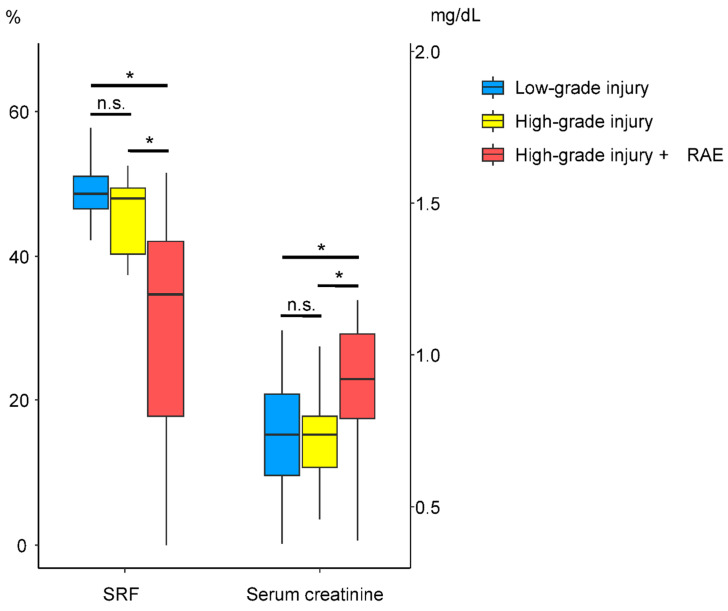
Comparison of SPECT SRF and serum creatinine according to AAST renal injury grade and RAE. * *p*-value < 0.05. n.s. = no significance.

**Figure 2:**
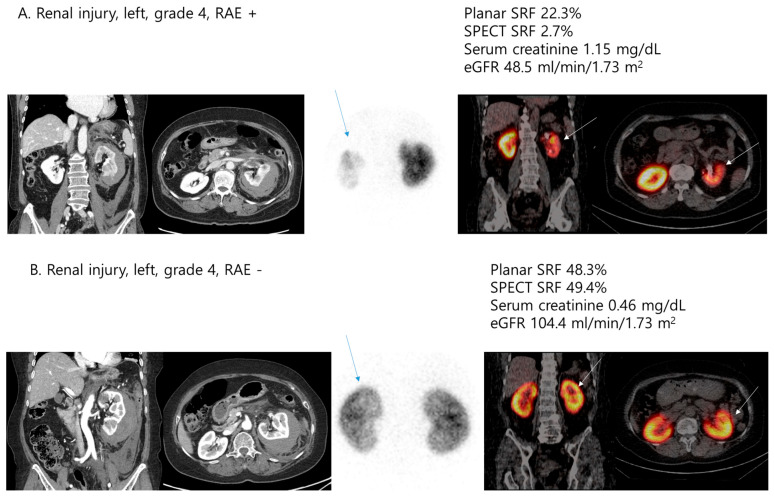
Comparison of clinical features (serum creatinine and eGFR) and SPECT SRF according to RAE (**A**,**B**).

The comparison of clinical features and DMSA scintigraphic parameters according to the performance of RAE is described in Table 5. Patients who underwent RAE showed significantly higher serum creatinine (0.74 ± 0.21 vs. 0.94 ± 0.30; *p* = 0.003) and lower GFR (108.20 ± 31.24 vs. 84.03 ± 23.43; *p* = 0.004) levels at follow-up, and significantly lower planar SRF (47.87 ± 8.48 vs. 37.61 ± 19.90; *p* = 0.036), SPECT SRF (47.41 ± 8.73 vs. 29.98 ± 17.28; *p* < 0.001), ICV (107.96 ± 33.47 vs. 65.77 ± 49.86; *p* = 0.002), ICU (13.12 ± 4.02 vs. 8.10 ± 6.49; *p* = 0.001), and ICV index values (61.65 ± 15.09 vs. 37.64 ± 27.90; *p* = 0.001). In addition, two simple graphical representations of the results in Table 3 and Table 5 revealed that patients with high-grade renal injury who underwent RAE had the highest serum creatinine levels and the lowest SPECT SRF, as shown in Figure 1 and Figure 2.

The predictive factors associated with significantly decreased residual cortical function are presented in Table 6. Univariate analyses revealed iatrogenic renal injury, high-grade renal injury, and RAE as significant factors (*p* = 0.025, *p* < 0.001, and *p* < 0.001, respectively). Moreover, the multivariate analysis illustrated that high grade renal injury (odds ratio (OR), 9.50; 95% confidence interval (CI), 1.78–50.61; *p* = 0.008) and RAE (OR, 5.15; 95% CI, 1.07–24.88; *p* = 0.041) were significant factors associated with decreased residual cortical function.

## 4. Discussion

This prospective observational study was aimed to assess residual cortical functions at follow-up in patients with renal injuries who underwent NOM treatment using Tc-99m DMSA SPECT, revealing that DMSA SPECT provides accurate information on the residual cortical function at follow-up in patients with renal injuries. In addition, the present study showed that high-grade renal injury and RAE are associated with decreased residual cortical function.

Traumatic renal injury is the most common urogenital trauma, accounting for 65% to 90% of all cases [2]. Meanwhile, the mechanisms of renal injury are diverse, and blunt abdominal trauma is responsible for 80% of renal injuries according to recent published renal trauma studies [1], which is similar to our study (74.6%). In addition, renal injuries are often accompanied by concomitant organ injuries, such as liver, spleen, adrenal gland, pancreas and intestine [1], probably because blunt trauma represents the most common cause of renal injury. In the present study, 44.1% of all patients had concomitant organ injuries, mainly involving the liver and spleen, depending on the site of renal injury. However, renal injuries from blunt trauma in polytraumatized patients were primarily low-grade injuries. In contrast, iatrogenic renal injuries were usually associated with isolated and high-grade injuries, which is probably due to the retroperitoneal location of the kidney.

As mentioned above, traumatic renal injury has a wide range of clinical outcomes due to diverse mechanisms of injury. Mortality in patients with renal injury was 5.5% to 13.4%, with various early and late complications such as bleeding, abscesses, hypertension, infections, arteriovenous fistulae and hypertension. [1] The management of renal injuries can be divided into non-operative (conservative or RAE) and operative approaches; most decisions are determined by hemodynamic stability and the grade of renal injury. Recently, the trend of renal injury treatment has undergone a paradigm shift toward NOM, which has become the standard treatment in the majority of Grade 1–4 injuries, even in patients with hemodynamically unstable high-grade renal injuries without evidence of other major visceral injuries requiring surgical exploration [2,4,5,19]. In addition, the improved grading of renal injury (e.g., AAST grade) and advancements in radiological techniques have increased the use of NOM [20]. Notably, RAE has assumed a pivotal role in the management of moderate to high-grade renal injury, with a successful rate of up to 94.9% for Grade 3, 89% for Grade 4, and 76% for Grade 5 injuries [9,21,22,23,24]. As a whole, primary NOM with or without RAE is associated with a higher rate of renal preservation without increasing the short-term or long-term morbidity [9]. In the present study, all patients who underwent RAE achieved clinical success at initial follow-up, although one patient underwent salvage nephrectomy for a symptomatic non-function kidney at 1 year after renal injury.

Despite the efficacy of RAE as a notable treatment option for NOM, this procedure is inevitably accompanied by some risks, including renal parenchymal infarction and contrast agent nephrotoxicity [7], which may lead to damage to renal function in the follow-up period. Therefore, the patients with high-grade renal injury, especially those who underwent RAE, may have concerns about the residual renal function. In several studies, renal function after traumatic renal injuries and associated treatments has been assessed using laboratory tests, primarily based on serum creatinine and GFR levels [6,8,9,10]. However, laboratory tests cannot precisely investigate the quantitative assessment of post-traumatic residual renal cortical function. Thus, imaging modalities, such as computed tomography (CT) and nuclear medicine scans, are employed to identify parenchymal infarction. In a study conducted by Chatziioannou et al., the post-embolisation parenchymal ischemic area, estimated by CT, ranged from 0 to 30% (mean 12%) in six consecutive patients who underwent embolisation for renovascular injuries [25]. However, CT scans also cannot provide accurate information for the quantitative assessment of post-traumatic residual renal cortical function at long-term follow-up. In fact, in the case of high-grade renal injury, although Hagiwara et al. found a similar decrease in the low density of localised renal areas in the first weeks after RAE in 18 trauma patients, these areas disappeared completely within 3 months of injury in all patients [26]. Meanwhile, nuclear medicine scans can be useful for assessing quantitative renal function after renal trauma injury and reconstruction. Currently, efforts have been made to quantitatively evaluate post-traumatic residual renal cortical function using Tc-99m DMSA scintigraphy. According to the results of these studies, post-traumatic residual renal cortical function ranged from 34% to 44% and varied depending on the distribution of renal injury grade [11,12,13].

Although DMSA scintigraphy can provide relatively accurate information on residual functional integrity, it has a limitation of depending on the SRF from the planar scan. SRF from planar scan can only detect a relative decrease in cortical integrity of the injured kidney compared to the contralateral, uninjured kidney; it does not describe the absolute amount of residual functional cortex after renal injury [15]. Hence, DMSA scintigraphy may be inappropriate in the follow-up of bilateral renal injuries or patients with underlying renal diseases (e.g., renal cysts or tumours) in the uninjured kidney. Conversely, DMSA SPECT has the advantage of improved image resolution and quantification, providing 3D volumetric indices and quantitative parameters of renal cortical integrity [14]. Our previous study on the additional value of DMSA SPECT over planar imaging found that SPECT SRF was significantly lower than planar SRF (mean bias of 2.3%), with particularly higher biases in cases of severe renal injury [15]. In the present study, the mean bias between planar SRF and SPECT SRF was 2.9%, with significantly lower SRF in SPECT. Additionally, we measured additional scintigraphic parameters from DMSA SPECT, including ICV, ICU, and ICV index, to calculate the delineated renal cortex of the injured kidney; these factors showed the best correlation with the renal injury grade. Based on the results of the present study, DMSA SPECT can provide accurate information on the residual cortical function at follow-up in patients with renal injuries who underwent NOM.

In the present study, patients with high-grade renal injury showed significantly higher serum creatinine and significantly lower SRF (both planar and SPECT) and additional SPECT scintigraphic parameters 3 months after the time of renal injury. Previous studies have likewise highlighted the correlation between AAST grade and renal function. Tasian et al. reported that the mean decrease in renal function for Grades 4 and 5 renal injuries was 30% and 65%, respectively, corresponding to relative renal function decreases of 35% and 17%, respectively [27]. In a study conducted by Fiard et al., the mean relative renal function for Grade 4 and 5 injuries was 39% and 11%, respectively [12]. These results are comparable to those in the present study. Moreover, high-grade renal injury was an independent predictor of significantly decreased residual cortical function in the present study. Other factors related to the patient (age, BMI, or gender) or the trauma (cause or side), as well as concomitant organ injuries, were not found to be associated with a decrease in residual cortical function. Considering these results, the AAST classification is particularly important for predicting the outcome of renal injury in terms of renal function independently of patient or trauma characteristics.

The present study included 20 patients who underwent RAE to manage renal injuries. Similarly to the high-grade renal injury, RAE was an independent predictive factor associated with significantly decreased residual cortical function, as well as significantly higher serum creatinine and lower DMSA scintigraphic parameters 3 months after the time of renal injury. In other previous studies, Xu et al. and Saour et al. reported no significant decrease in renal function after embolisation, while Vozianov reported two patients (10%) who exhibited a significant reduction in renal function at the 3-month follow-up [6,28,29]. These discrepancies may exist depending on related factors such as the depth of embolisation, selectivity, and the nature of the embolic agent. In summary, when performing RAE, it is necessary to have accurate indications and explain to the patient the possibility of decreased residual cortical function.

The present study has limitations. First, this small-scale study only included patients from a single institution, may have some selection bias. Furthermore, the follow-up period of the present study is relatively short; we assessed residual cortical function in patients with renal injuries using DMSA SPECT at 3 months follow-up and evaluated clinical factors associated with decreased residual cortical function. However, there was no additional deterioration in patients with high-grade injury 1 year after the time of renal injury; regular DMSA follow-up may not be necessary. Second, although SPECT SRF provides more accurate information on residual cortical function than planar SRF at follow-up in patients with renal injuries, it inevitably accompanies limitations. For example, SRF may discount other factors related to patients (e.g., difference in native size, compensatory hypertrophy of the uninjured kidney, and the patient`s body profile) [15]. Hence, we measured additional scintigraphic parameters from DMSA SPECT, such as ICV, ICU and ICV index values, to compensate for the limitation of SRF; these factors provide the volume of the delineated renal cortex of the injured kidney. Lastly, the effect of RAE on residual cortical function in patients with renal injury may be biassed depending on the depth of embolisation selectivity and the kind of embolic agent. In the present study, most patients (85%) underwent superselective angioembolization, and it was performed using a microcoil to maximise the preservation of renal parenchyma. This suggests that the present study revealed the effect of RAE on residual cortical function following renal injury.

To our knowledge, the present study is the first prospective observational study to assess residual cortical function at follow-up in non-operatively treated patients with renal injuries using Tc-99m DMSA SPECT. Considering the results of this study, DMSA SPECT may be an important modality for follow-up in traumatic renal injury. Additionally, we recommend considering DMSA SPECT for monitoring renal function after renal injury. Although a large-scale longitudinal study is needed, our results can provide evidence for future guidelines of renal trauma.

## 5. Conclusions

Traumatic renal injury is the most common urogenital trauma, with a wide range of clinical outcomes. Therefore, patients with renal injury may have concerns about the residual renal function. In this context, the present study revealed that DMSA SPECT provide additive, clinically relevant, and comprehensive information on the residual cortical function at follow-up in patients with renal injuries. In addition, the patients with high-grade renal injury, especially those who underwent RAE, were significantly associated with decreased residual cortical function. Therefore, it is necessary to have accurate indications for RAE and explain to the patient the possibility of decreased residual cortical function. In summary, DMSA SPECT may serve as an important modality for assessing residual cortical integrity, and will be included in future guidelines for follow-ups after renal trauma.

## Figures and Tables

**Table 1:** Demographic characteristics of patients with renal injury.

Variables	n = 59
Age (years)	49.10 ± 22.67
BMI (kg/m^2^)	24.24 ± 4.22
Sex	
Male	35 (59.3%)
Female	24 (40.7%)
Comorbidities	
Hypertension	19 (32.2%)
Diabetes mellitus	8 (13.6%)
Underlying renal diseases	
Cyst	16 (27.1%)
Others	10 (16.9%)
Causes of renal injuries	
Traffic accident	23 (39.0%)
Fall-down	12 (20.3%)
Slip-down	9 (15.3%)
Iatrogenic	12 (20.3%)
Others	3 (5.1%)
Injured kidney	
Right	24 (40.7%)
Left	35 (59.3%)
AAST grades	
1	6 (10.2%)
2	10 (16.9%)
3	15 (25.4%)
4	23 (39.0%)
5	5 (8.5%)
Concomitant organ injuries	26 (44.1%)
Renal artery endovascular procedure	20 (33.9%)
Renal function	
At index injury	
Serum creatinine (mg/dL)	0.97 ± 0.39
eGFR (mL/min/1.73 m^2^)	88.87 ± 32.65
Follow-up	
Serum creatinine (mg/dL)	0.81 ± 0.26
eGFR (mL/min/1.73 m^2^)	100.00 ± 30.86
Tc-99m DMSA scintigraphy	
Planar SRF (%)	44.39 ± 14.17
SPECT SRF (%)	41.50 ± 14.73
ICV (mL)	93.65 ± 44.21
ICU (%)	11.42 ± 5.49
ICV index, mL/m^2^	53.51 ± 23.14

BMI: body mass index; AAST: American Association for the Surgery of Trauma; eGFR: estimated glomerular filtration rate; DMSA: dimercaptosuccinic acid; SRF: split renal function; SPECT: single-photon emission computed tomography; ICV: injured renal cortical volume; ICU: injured renal cortical uptake (absolute % injected dose). SRF values were measured for the injured kidney. Data are presented as the mean ± standard deviation for continuous variables and number (%) for categorical variables.

**Table 2:** Correlations between kidney injury grade and clinical features, as well as DMSA scintigraphic parameters.

	AAST Renal Injury Grade	
	Correlation Coefficients	*p*-Value
Age (years)	0.247	0.059
BMI (kg/m^2^)	−0.156	0.239
Serum creatinine (mg/dL) at follow-up	0.290	0.026
eGFR (mL/min/1.73 m^2^) at follow-up	−0.336	0.009
Concomitant organ injuries	−0.266	0.042
Planar SRF (%)	−0.291	0.025
SPECT SRF (%)	−0.565	<0.001
ICV (mL)	−0.551	<0.001
ICU (%)	−0.450	<0.001
ICV index (mL/m^2^)	−0.555	<0.001

BMI: body mass index; AAST: American Association for the Surgery of Trauma; eGFR: estimated glomerular filtration rate; DMSA: dimercaptosuccinic acid; SRF: split renal function; SPECT: single-photon emission computed tomography; ICV: injured renal cortical volume; ICU: injured renal cortical uptake (absolute % injected dose). SRF values were measured for the injured kidney. Correlation coefficients are based on Pearson’s correlation analysis.

**Table 3:** Comparison of clinical features and DMSA scintigraphic parameters according to the AAST renal injury grade.

Variable	Low Grade Injuries (n = 31)	High Grade Injuries (n = 28)	*p*-Value
Age (years)	46.39 ± 24.45	52.11 ± 20.56	0.370 ^a^
BMI (kg/m^2^)	24.63 ± 5.01	23.81 ± 3.16	0.451 ^b^
Sex			0.461 ^c^
Male	17 (54.8%)	18 (64.3%)	
Female	14 (45.2%)	10 (35.7%)	
Hypertension	10 (32.3%)	9 (32.1%)	0.992 ^c^
Diabetes mellitus	4 (12.9%)	4 (14.3%)	1.000 ^d^
Causes of renal injuries			0.053 ^d^
Traffic accident	16 (51.6%)	7 (25.0%)	
Fall-down	8 (25.8%)	4 (14.3%)	
Slip-down	3 (9.7%)	6 (21.4%)	
Iatrogenic	3 (9.7%)	9 (32.1%)	
Others	1 (3.2%)	2 (7.1%)	
Injured kidney			0.461 ^c^
Right	14 (45.2%)	10 (35.7%)	
Left	17 (54.8%)	18 (64.3%)	
Concomitant organ injuries	16 (51.6%)	10 (35.7%)	0.219 ^c^
Renal artery endovascular procedure	1 (3.2%)	19 (67.9%)	<0.001 ^d^
Renal function at index injury			
Serum creatinine (mg/dL)	0.87 ± 0.27	1.09 ± 0.47	0.104 ^a^
eGFR (mL/min/1.73 m^2^)	96.69 ± 32.29	80.20 ± 31.36	0.052 ^b^
Renal function at follow-up			
Serum creatinine (mg/dL)	0.74 ± 0.19	0.89 ± 0.30	0.024 ^a^
eGFR (mL/min/1.73 m^2^)	107.28 ± 30.71	91.95 ± 29.49	0.056 ^b^
DMSA scintigraphy			
Planar SRF (%)	48.75 ± 8.24	39.57 ± 17.61	0.022 ^a^
SPECT SRF (%)	48.91 ± 6.38	33.30 ± 16.97	<0.001 ^a^
ICV (mL)	113.07 ± 30.69	72.15 ± 47.32	<0.001 ^b^
ICU (%)	13.41 ± 3.38	9.22 ± 6.52	0.004 ^b^
ICV index (mL/m^2^)	64.58 ± 12.55	41.27 ± 26.06	<0.001 ^b^

BMI: body mass index; AAST: American Association for the Surgery of Trauma; eGFR: estimated glomerular filtration rate; DMSA: dimercaptosuccinic acid; SRF: split renal function; SPECT: single-photon emission computed tomography; ICV: injured renal cortical volume; ICU: injured renal cortical uptake (absolute % injected dose). SRF values were measured for the injured kidney. Data are presented as the mean ± standard deviation for continuous variables and number (%) for categorical variables. ^a^: Wilcoxon rank-sum test; ^b^: Student’s *t*-test; ^c^: Pearson chi-square test; ^d^: Fisher’s exact test.

**Table 4:** Comparison of clinical features and DMSA scintigraphic parameters in patients with high-grade renal injury according to follow-up period.

Variable	3 Months	1 Year	*p*-Value
Renal function			
Serum creatinine (mg/dL)	0.89 ± 0.35	0.88 ± 0.24	0.346 ^a^
eGFR (mL/min/1.73 m^2^)	97.22 ± 35.00	95.03 ± 31.63	0.337 ^b^
DMSA scintigraphy			
Planar SRF (%)	43.28 ± 14.22	41.82 ± 15.29	0.331 ^b^
SPECT SRF (%)	35.55 ± 16.58	35.65 ± 15.69	0.798 ^a^
ICV (mL)	73.17 ± 49.33	75.14 ± 49.25	0.719 ^b^
ICU (%)	9.45 ± 7.08	10.21 ± 6.44	0.360 ^b^
ICV index (mL/m^2^)	42.63 ± 26.38	44.35 ± 27.30	0.563 ^b^

eGFR: estimated glomerular filtration rate; DMSA: dimercaptosuccinic acid; SRF: split renal function; SPECT: single-photon emission computed tomography; ICV: injured renal cortical volume; ICU: injured renal cortical uptake (absolute % injected dose). SRF values were measured for the injured kidney. Data are presented as the mean ± standard deviation for continuous variables. ^a^: Wilcoxon signed rank-sum test; ^b^: paired *t*-test.

**Table 5:** Comparison of clinical features and DMSA scintigraphic parameters according to renal artery endovascular procedure.

Variable	No (n = 39)	Yes (n = 20)	*p*-Value
Age (years)	44.74 ± 23.51	57.60 ± 18.69	0.040 ^a^
BMI (kg/m^2^)	24.40 ± 4.84	23.91 ± 2.69	0.620 ^b^
Sex			0.525 ^c^
Male	22 (56.4%)	13 (65.0%)	
Female	17 (43.6%)	7 (35.0%)	
Hypertension	9 (23.1%)	10 (50.0%)	0.036 ^c^
Diabetes mellitus	5 (12.8%)	3 (15.0%)	1.000 ^d^
Causes of renal injuries			0.255 ^d^
Traffic accident	18 (46.1%)	5 (25.0%)	
Fall-down	9 (23.1%)	3 (15.0%)	
Slip-down	4 (10.3%)	5 (25.0%)	
Iatrogenic	6 (15.4%)	6 (30.0%)	
Others	2 (5.1%)	1 (5.0%)	
Injured kidney			0.232 ^c^
Right	18 (46.1%)	6 (30.0%)	
Left	21 (53.9%)	14 (70.0%)	
Concomitant organ injuries	19 (48.7%)	7 (35.0%)	0.315 ^c^
Renal function at index			
Serum creatinine (mg/dL)	0.91 ± 0.37	1.10 ± 0.41	0.058 ^a^
eGFR (mL/min/1.73 m^2^)	96.59 ± 34.07	73.81 ± 23.89	0.010 ^b^
Renal function at follow-up			
Serum creatinine (mg/dL)	0.74 ± 0.21	0.94 ± 0.30	0.003 ^a^
eGFR (mL/min/1.73 m^2^)	108.20 ± 31.24	84.03 ± 23.43	0.004 ^b^
DMSA scintigraphy			
Planar SRF (%)	47.87 ± 8.48	37.61 ± 19.90	0.036 ^a^
SPECT SRF (%)	47.41 ± 8.73	29.98 ± 17.28	<0.001 ^a^
ICV (mL)	107.96 ± 33.47	65.77 ± 49.86	0.002 ^b^
ICU (%)	13.12 ± 4.02	8.10 ± 6.49	0.001 ^b^
ICV index (mL/m^2^)	61.65 ± 15.09	37.64 ± 27.90	0.001 ^b^

BMI: body mass index; eGFR: estimated glomerular filtration rate; DMSA: dimercaptosuccinic acid; SRF: split renal function; SPECT: single-photon emission computed tomography; ICV: injured renal cortical volume; ICU: injured renal cortical uptake (absolute % injected dose). SRF values were measured for the injured kidney. Data are presented as the mean ± standard deviation for continuous variables and number (%) for categorical variables. ^a^: Wilcoxon rank-sum test; ^b^: Student’s *t*-test; ^c^: Pearson chi-square test; ^d^: Fisher’s exact test.

**Table 6:** Clinical factors associated with significantly decreased residual cortical function (SRF < 45%).

Variables	Univariate Analysis		Multivariate Analysis	
	Odds Ratio (95% CI)	*p*-Value	Odds Ratio (95% CI)	*p*-Value
Age (years)	1.02 (0.99–1.04)	0.129	Not applicable	
BMI (kg/m^2^)	1.03 (0.91–1.17)	0.605		
Sex				
Male	Reference			
Female	1.21 (0.42–3.50)	0.726		
Causes of renal injuries				
Traffic accident	Reference			
Fall-down	0.94 (0.19–4.70)	0.944		
Slip-down	3.54 (0.71–17.73)	0.124		
Iatrogenic	5.67 (1.24–25.88)	0.025		
Others	1.42 (0.11–18.59)	0.791		
Injured kidney				
Right	Reference			
Left	2.05 (0.68–6.16)	0.204		
AAST renal injury grades				
Low	1 (Reference)		1 (Reference)	
High	23.33 (5.50–99.04)	<0.001	9.50 (1.78–50.61)	0.008
Concomitant organ injuries	0.72 (0.25–2.08)	0.542		
Renal artery endovascular procedure	18.29 (4.66–71.76)	<0.001	5.15 (1.07–24.88)	0.041

BMI: body mass index; AAST: American Association for the Surgery of Trauma; SRF: split renal function; SRF values were measured for the injured kidney.

## Data Availability

The data presented in this study are available in this article.
